# Lymphocytic Choriomeningitis Virus–associated Meningitis, Southern Spain

**DOI:** 10.3201/eid1805.111646

**Published:** 2012-05

**Authors:** Mercedes Pérez-Ruiz, José-María Navarro-Marí, María-Paz Sánchez-Seco, María-Isabel Gegúndez, Gustavo Palacios, Nazir Savji, W. Ian Lipkin, Giovanni Fedele, Fernando de Ory-Manchón

**Affiliations:** Hospital Universitario Virgen de las Nieves, Granada, Spain (M. Pérez Ruiz, J.-M. Navarro-Mari);; Instituto de Salud Carlos III, Majadahonda, Madrid, Spain (M.-P. Sánchez-Seco, G. Fedele, F. de Ory-Manchón);; Universidad de Alcalá, Alcalá de Henares, Spain (M.-I. Gegúndez);; Columbia University, New York, New York, USA (G. Palacios, N. Savji, W.I. Lipkin)

**Keywords:** Lymphocytic choriomeningitis virus, meningitis, Spain, encephalitis, viruses

## Abstract

Lymphocytic choriomeningitis virus (LCMV) was detected in 2 patients with acute meningitis in southern Spain within a 3-year period. Although the prevalence of LCMV infection was low (2 [1.3%] of 159 meningitis patients), it represents 2.9% of all pathogens detected. LCMV is a noteworthy agent of neurologic illness in immunocompetent persons.

Lymphocytic choriomeningitis virus (LCMV; family *Arenaviridae*) is a rodent-borne pathogen; its main reservoir is the common house mouse (*Mus musculus*), but it has also been detected in pets, research rodents, and wild mice (*1**,**2*). Presumed transmission routes to humans are ingestion or inhalation of contaminated rodent feces, urine, or both. Although LCMV usually produces a self-limited mild or asymptomatic infection, it can cause aseptic meningoencephalitis (AME) with teratogenic effects in infants (*3*). Although LCMV was one of the earliest viruses to be associated with human AME, it is now rarely reported as an etiologic agent (*4*).

Historically, in Spain, LCMV was detected in 1 person with encephalitis (*5*) and 4 persons with meningitis (*6*). A similar virus, characterized as LCMV lineage IV, was identified in rodents (*2*).

During 2008–2010, a multicenter project was conducted to investigate viral causes of AME in Spain. The following viruses were considered: human enterovirus (HEV), herpesviruses, Toscana virus (TOSV), mumps virus, phleboviruses, flaviviruses, arenaviruses, and adenoviruses. We report 2 LCMV meningitis case-patients who lived 1,200 meters apart within Granada Province in southern Spain.

## The Study

The study period was January 2008–June 2010. The population included patients at Hospital Virgen de las Nieves (Granada, Spain) who had suspected AME. Routine virologic investigation included reverse transcription PCR (RT-PCR) of cerebrospinal fluid (CSF) samples for detection of HEV (Xpert EV system, Cepheid, Sunnyvale, CA, USA), TOSV (*7*), and mumps virus (*8*), and PCR of herpes simplex virus (HSV) and varicella-zoster virus (LC VHS 1/2 Qual and LC VZV systems, respectively; Roche Diagnostics, Mannheim, Germany). Negative samples were subsequently tested for Epstein-Barr virus, cytomegalovirus, human herpes 6 virus (*9*), flavivirus (*10*), arenavirus (*2*), adenovirus (*11*), and phlebovirus (*12*). Specific LCMV RT-PCR was conducted by using a previously described protocol (*2*). Finally, CSF specimens from PCR-negative case-patients were inoculated in Vero and MRC-5 cell lines for viral culture.

CSF and acute-phase serum samples were also tested for IgG and/or IgM against TOSV (EIA Enzywell Toscana virus IgG/IgM, Diesse, Siena, Italy), West Nile virus (ELISA IgG and IgM-capture ELISA; Focus Diagnostics, Cypress, CA, USA), and LCMV by indirect fluorescent assay (IFA) with further confirmation by Western blot (*2*).

We studied 159 CSF samples by using PCRs for the presence of HEV, HSV, varicella-zoster virus, TOSV, and mumps virus, yielding 68 positive cases. The remaining viruses were further investigated in the 91 negative samples. A viral agent was detected in 70 (44%) cases: HEV accounted for 44 (63%) of positive cases, followed by varicella-zoster virus in 11 (16%), HSV-1 in 8 (11%), TOSV in 4 (6%), LCMV in 2 (3%), and HSV-2 in 1 (1%).

**Figure 1:**
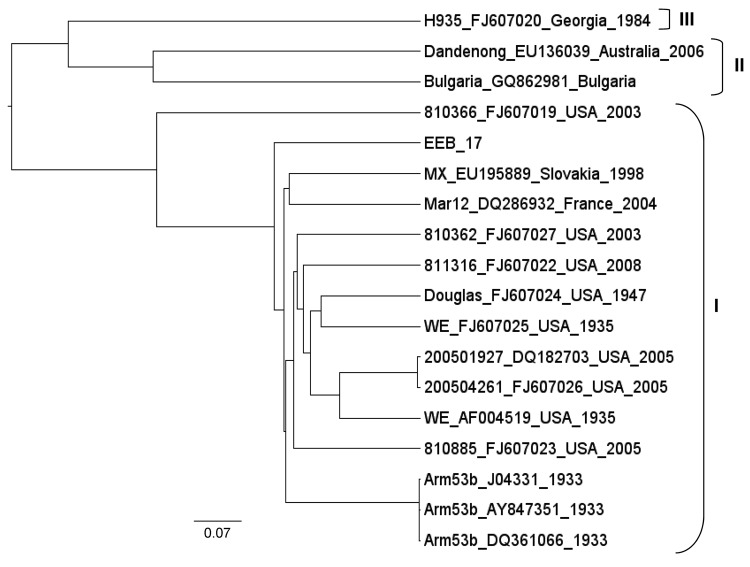
Phylogenetic tree showing genetic lymphocytic choriomeningitis virus sequences relationship within the large segment. The name of the strain is followed by GenBank accession number, country, and year of detection. Clusters grouped in brackets depict the lymphocytic choriomeningitis virus lineage. Scale bar indicates nucleotide substitutions per site.

**Figure 2:**
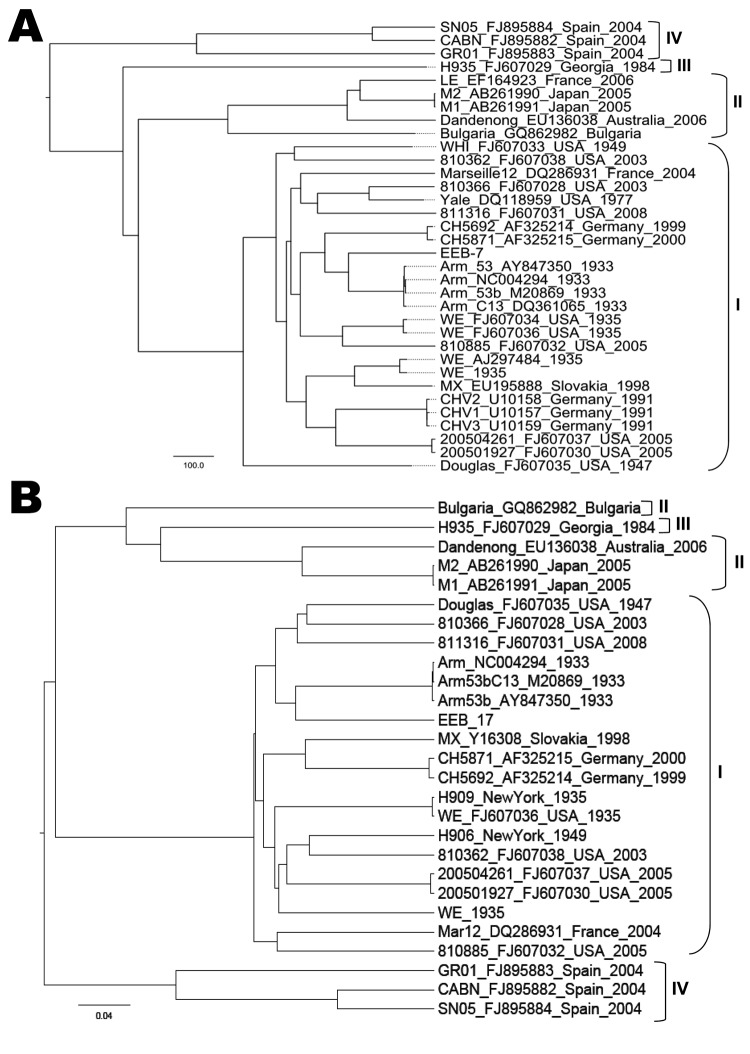
Phylogenetic tree showing genetic lymphocytic choriomeningitis virus sequences relationship within the small segment coding for the glycoprotein complex (A) and nucleocapsid proteins (B). The name of the strain is followed by GenBank accession number, country, and year of detection. Clusters grouped in brackets depict the lymphocytic choriomeningitis virus lineage. Scale bars indicate nucleotide substitutions per site.

Case-patient 1, a 21-year-old woman, came to the hospital’s emergency unit in April 2008 exhibiting headache, chills, fever (38.9°C), confusion, nausea, vomiting, and slight nuchal rigidity. Aseptic meningitis was suspected, and samples of CSF and serum were obtained. Laboratory results were 415 leukocytes/mm^3^ (100% mononuclear cells), 43 mg/100 dL glucose level (reference 35–65 mg/dL), and 128 mg/dL protein level (reference 15–45 mg/dL) in the CSF. Results of a computed tomographic scan of the brain were normal. IgG and IgM titers of 640 and 128 against LCMV were detected in the serum sample by IFA and titers of 400 and 200 by Western blot assay, respectively. Results of RT-PCR for arenavirus and LCMV were negative. Viral culture of the CSF in Vero cells revealed no cytopathic effect after 1 month of incubation. Cell culture supernatants from several passages were subjected to specific RT-PCR. LCMV PCR was positive at a dilution of 10^−4^ at the third passage. Viral isolate (EEB-7) was used for genetic characterization. To sequence the genome, degenerate and specific primers were designed on the basis of an alignment of all complete LCMV large (L), glycoprotein complex and nucleocapsid protein sequences (Table).Terminal sequences were generated by using a universal arenavirus primer, targeting the conserved viral termini (5′-CGC ACM GDG GAT CCT AGG C-3′), combined with 4 specific primers positioned near the ends of each segment. Amplification products were size-fractionated by electrophoresis in 1% agarose gels, purified (MinElute, QIAGEN, Valencia, CA, USA), and sequenced in both directions on an ABI PRISM 3700 DNA analyzer (PE Applied Biosystems, Foster City, CA, USA). To determine the evolutionary history of isolate EEB-7, we performed Bayesian phylogenetic analysis of all available complete genome sequences of LCMV using BEAST, BEAUti, and Tracer analysis software packages (*13*). The analysis showed that EEB-7 (GenBank accession nos. JN872494–5) belonged to LCMV lineage I (Figure 1, Figure 2). Clinical diagnosis was acute meningitis and the patient was discharged from hospital on day 9.

**Table:** Consensus primers used to identify LCMV sequences, southern Spain, 2008–2010*

Name	Sequence, 5′ → 3′
LCMV_L_1F	ATAAARTGYTTTGARAARTTYTTTGA
LCMV_L_1R	TCAAARAAYTTYTCAAARCAYTTTAT
LCMV_L_2R	CATYTTCAWRCAWGARAACCAATC
LCMV_L_3R	CCWGARTABGMRTTNGCATTCAT
LCMV_L_2F	AGYAARTGGGGNCCVATGATGTG
LCMV_L_3F	GARGTHCCHTTYCCTGTTGT
LCMV_L_4F	TCYAGYCTYATTGAYATGGG
LCMV_L_5F	AARTTYACHAGRGGNGCRCAGAA
LCMV_L_4R	GTRTARTGYTCRTCYCTYTTCCA
LCMV_L_5R	CCRTCYTCDGANGGCATCAT
LCMV_L_6F	GGNTWYGGDTGGTTYTCTTA
LCMV_L_6R	GGSACDGGBTCCCABTCAGG
LCMV_GPC_1R	GARCARGARGCVGAYAAYATGAT
LCMV_GPC_1F	TARTTRCARTANGGYACNCCCAT
LCMV_GPC_2F	CCNCCRAARGCWGTYCTRAACAT
LCMV_GPC_2R	AGRGGNAGRGTBYTRGAYATGTT
LCMV_GPC_3R	TCYCAYCAYTAYATHAGYATGGG
LCMV_NP_1F	ATGCCVAGYYTRACHATGGC
LCMV_NP_1R	GCCATDGTYARRCTBGGCAT
LCMV_NP_2F	GAYGTYGTRCARGCVCTMACAGA
LCMV_NP_2R	CCHACYTGRTCNGADACRAACAT

*LCMV, lymphocytic choriomeningitis virus; GPC, glycoprotein comlex; NP, nucleocapsid protein.

Case-patient 2, a 39-year-old man, sought treatment in May 2010 with headache, nausea, vomiting, increased perspiration, and a temperature of 37.5°C. Aseptic meningitis was suspected, and CSF and serum samples were collected. CSF analysis demonstrated 1,715 leukocytes/mm^3^ (95% mononuclear cells), normal glucose level (68 mg/dL), and elevated protein levels (240 mg/dL). Results of a cranial computed tomographic scan were normal. Further virologic investigation detected LCMV RNA in the CSF and an IgG titer of 640 by IFA and IgM antibodies against LCMV in the serum sample. Serum amount was insufficient to conduct IgM titration and Western blot assay. No CSF sample was available to attempt viral isolation. The sequence of a 194-nt PCR product (nucleocapsid protein gene) obtained was most closely related to sequences of the lineage I. Sequence homology among the LCMV amplicon from case-patient 2 and lineage I strains was >87% versus 77%–79% sequence homology among case-patient 2 and strains from lineages II–IV. Clinical diagnosis was subacute meningitis, and the patient was discharged on day 16.

PCR has become the reference standard for identifying common viruses involved in AME (*6*). However, no commercial tests are available for LCMV; and in-house PCRs have to be optimized according to the natural genetic diversity of the virus (*14*). Serologic testing has been found useful in detecting LCMV. Neurologic LCMV infection in Spain has been diagnosed primarily by serologic tests (*5**,**6*). The diagnosis in case-patients 1 and 2 in the current study was achieved by serologic testing as well. Nonetheless, PCR was useful because it allowed genetic characterization of the LCMV strain from case-patient 2. Furthermore, the viral isolate of case-patient 1 was evident only by detection of LCMV RNA in the cell culture supernatant because no cytopathic effect was observed.

Isolates from both LCMV case-patients belonged to the classical lineage I, which has been detected elsewhere in Europe. Lineage I is usually associated with human disease (as are lineages II and III) and is linked to the common house mouse as its reservoir (*14*). Lineage IV viruses were previously detected in Spanish wood mice (*2*) and have not been associated with human disease.

Although no further epidemiologic studies could be conducted to search for infected reservoirs, the common genetic lineage and the fact that both case-patients resided within the same community might suggest transmission of LCMV by a common vector. Previous seroprevalence studies have associated human LCMV infection with high exposure to the common house mouse (*15*).

## Conclusions

Human LCMV infections might be underdiagnosed because the clinical characteristics of LCMV meningitis are similar to those of other viral meningitis; no commercial tests are available for serologic or molecular diagnostic assays, and usually no clear epidemiologic clue is available at the moment of diagnosis. Thus, epidemiologic and virologic surveillance might ascertain that the true incidence of LCMV AME is more frequent than reported.
